# Bisphosphonates, vitamin D, parathyroid hormone, and osteonecrosis of the jaw. Could there be a missing link?

**DOI:** 10.4317/medoral.20927

**Published:** 2016-01-31

**Authors:** Ignacio-Osoitz Leizaola-Cardesa, Antonio Aguilar-Salvatierra, Maximino Gonzalez-Jaranay, Gerardo Moreu, María-José Sala-Romero, Gerardo Gómez-Moreno

**Affiliations:** 1 Department of Pharmacological Research in Dentistry, Master of Periodontology and Implant Dentistry, Faculty of Dentistry, Universidad de Granada, Granada, Spain; 2 Department of Periodontology, Director of Master of Periodontology and Implant Dentistry, Faculty of Dentistry, University of Granada, Granada, Spain; 3 Private practice in Endocrinology, Granada, Spain; 4 Department of Special Care in Dentistry, Pharmacological Research in Dentistry, Director of Master of Periodontology and Implant Dentistry, Faculty of Dentistry, University of Granada, Granada, Spain

## Abstract

It is estimated that over 190 million bisphosphonates have been prescribed worldwide. But this drug can produce adverse effects, of which osteonecrosis of the jaw and severe hypocalcemia are the most serious. It is evident that bisphosphonate administration affects multiple and diverse biochemical mediators related to bone metabolism. This review of literature investigates four basic parameters in patients treated with bisphosphonates - parathyroid hormone (PTH), bisphosphonates, vitamin D, calcium, and jaw osteonecrosis - which are fundamental for assessing bone metabolism and so the efficacy and correct use of the drug. The imbalances generated by vitamin D and calcium deficiencies, together with their multiple systemic repercussions, have been widely researched but the outcomes of these imbalances in relation to bisphosphonate administration are not well known, and some research has indicated that they may be associated with osteonecrosis of the jaw (ONJ). The present review set out to explain the functioning of bone metabolism, the importance of different chemical mediators, the imbalances produced by incorrect use of this drug, in order to forewarn against the possible relation of these parameters with ONJ, whose physiopathology remains unknown. Medical and dental clinics should keep detailed anamneses of the use of vitamin D and calcium supplements, as it is of vital importance to maintain their correct levels in blood, given that these are related to ONJ as well as other adverse effects; this procedure is also necessary in order to ensure the correct use of the drug.

** Key words:**Bisphosphonate-related osteonecrosis of the jaw, vitamin D, parathyroid hor

## Introduction

Bisphosphonates are potent inhibitors of osteoclast activation, and the first choice for treating pathologies that affect bone metabolism such as osteoporosis and Paget’s disease. They are also used to treat malignant tumors such as multiple myeloma or other tumors with bone metastasis such as prostate and breast cancers. According to the Advisory Task Force on Bisphosphonate-related Osteonecrosis of Jaws (2007), 190 million bisphosphonates have been prescribed worldwide (1,2). It is said that osteoporotic bone has a negative balance or lack of calcium so that traditionally it has been treated with vitamin D and calcium supplements, moderate exercise, and treatment with bisphosphonates in cases with a risk of fracture (3,4). Generic vitamin D refers to both vitamin D3 (physiological in humans) and vitamin D2 (ultraviolet radiation). When a vitamin D deficiency is produced, calcium absorption decreases by 15% (phosphorous absorption by 60%), reducing serum ionized calcium. This decrease is detected by parathyroid gland calcium sensors, which respond with an increase in parathyroid hormone (PTH) whose function is to maintain serum calcium levels, acting on the liver and on bone where it stimulates bone resorption.

When the calcium supply to the organism is inadequate, the vitamin D hormonally active metabolite (1,25 (OH)2D) helps maintain calcium homeostasis, acting on vitamin D receptors (VDR) of the osteoblasts in which receptor activator of nuclear factor kappa-B ligand (RANKL) formation is induced in a similar way as with PTH, so producing bone resorption for calcium homeostasis (3-5). When the major circulating vitamin D metabolite (25(OH)D) levels are very reduced a failure to produce correct calcium absorption in the intestine occurs, with insufficient substrate for converting 1,25(OH)2D, even when PTH levels are high (6,7).

For many years it was thought that the transformation of 25(OH)D into 1,25(OH)2D was produced only in the liver by the enzyme 25(OH)D-1α-hydroxylase but this enzyme has also been found in other tissues in the organism. On the basis of extensive evidence, it is understood that vitamin D is related to immune system regulation (8,9).

Vitamin D has other auto/paracrine functions throughout the organism. Most tissues and cells, whether normal or neoplastic - muscle, heart, brain, blood vessels, breast, colon, pancreas, skin, and the immune system – all have VDRs and 25(OH)D activating enzymes, which are not regulated by PTH to synthesize 1,25(OH)2D (3,4). 1,25(OH)2D attaches to its VDR and regulates the transcription of approximately 3% of the human genome. It also modulates macrophage and lymphocyte B and T function. For all these reasons, 1,25(OH)2D plays an important role in general health (3,4).

The optimal level of vitamin D has recently been described as follows: sufficient vitamin D concentration so that serum PTH levels are neither elevated nor decreased when vitamin D supplements are taken (10). PTH would appear to have a stimulating effect of bone destruction/formation through calcium homeostasis – endocrine actions that might be described as ‘traditional’ or ‘classic.’ It has been proved that increased PTH administered continuously has the effect of stimulating bone destruction. But intermittent administration stimulates bone formation (a process known as osteoformative or anabolic). The reasons for these differences remain unclear (3,4). PTH has a direct effect via diverse mechanisms that produce this increase in osteoblast numbers, stimulating their differentiation and inhibiting their apoptosis. Among the various osteoblast-stimulating factors, several proteins have been identified and also 1,25(OH)2D, which, after being synthesized in the liver under PTH stimulation, stimulate osteoblasts through endocrine action (3).

It has been indicated that hypovitaminosis D presents secondary hyperparathyroidism that increases bone renewal, losing bone mass, and in severe cases can lead to osteomalacia (10). The latest tendency in osteonecrosis treatment is the use of teriparatide (recombinant human PTH 1-34). One study describes how a patient with jaw osteonecrosis, having failed to respond to conventional approaches, was successfully treated with teriparatide (11).

The present review set out to explain the functioning of bone metabolism, the importance of different chemical mediators, the imbalances produced by incorrect use of this drug, in order to forewarn against the possible relation of these parameters with ONJ, whose physiopathology remains unknown.

## Material and Methods

This review was based on a literature search conducted in the following online databases.

1. Medline.

2. Embase.

Key search words were used to select texts: PTH, Bisphosphonates, Bisphosphonate-related osteonecrosis of the jaw (BORNJ), ONJ, Vitamin D and Calcium. The search also consulted the database Uptodate® which located additional texts. The inclusion criteria also included editorials, case control studies, retrospective studies and randomized double-blind studies related with bisphosphonates, vitamin D, parathyroid hormone, and osteonecrosis of the jaw. The search was not limited to human studies. We updtated publications with papers from January 1996 to May 2015. The articles identified were screened in duplicate (two reviewers, I.O.L.C. and A.A.S.). The full papers and abstracts identified were independently reviewed by the authors (I.O.L.C and A.A.S) for inclusion in this review of literature. If there was lack of information provided in the abstract or disagreement between the reviewers, the authors reviewed the full text before reaching an agreement. An endocrinologist (M.J.S.R.) monitored all the endocrine aspects. M.G.J. and G.M. supervised the dental an bone aspects. The review was directed and supervised by G.G.M.

## Results

The initial search identified 15 articles of which six did not fulfill the inclusion criteria, leaving eight articles and one editorial, all published between 1996 and 2013. Of the articles, four were case-control studies, three was a retrospective studies and one randomized double-blind studies (12-20).

The editorial selected posed the question of whether increases in PTH levels and hypocalcemia during bisphosphonate treatment in patients with metastatic breast cancer could be predisposing factors to osteonecrosis of the jaw. The article drew attention to the fact that secondary hyperparathyroidism after taking bisphosphonates may be related to ONJ, an issue that has never been investigated (12).

Three articles were found that deal with predictive PTH values for preventing NJO, although with contradictory results. A case-control study (13) found significantly larger increases in PTH among bisphosphonate patients with BRONJ than control subjects taking bisphosphonates but who did not suffer BRONJ. However, two other studies (14,15) did not find any significant relation between ONJ and PTH levels. None of these three studies took vitamin D and calcium supplementation into consideration.

Another study (16) compared Group A patients in treatment with Etidronate who showed vitamin D deficiency with Group B patients subject to the same treatment but presenting adequate vitamin D levels. Group A had significantly higher levels of PTH and lower serum calcium levels one year after the start of treatment with Etidronate. In the Group A base sample, PTH had shown normal values; secondary hyperparathyroidism was expected as a result of hypovitaminosis D.

A patient sample of post-menopausal women taking bisphosphonates and presenting increased PTH values were seen to obtain increased bone densitometry values, suggesting that the better the response to medication, the better the gains in bone mass. All the women took vitamin D and calcium supplements (17).

One study related increases in nocturnal PTH levels with bone mass gain (18). Patients treated with bisphosphonates present nocturnal peaks in PTH, which point to an anabolic effect of PTH. It is a well known fact that endocrine gland functions are related to circadian rhythm. All patients underwent rigorous management of serum calcium levels and were administered with supplements orally.

The following article (19) related hypovitaminosis D with an increased incidence of osteonecrosis in rats; the osteonecrosis induced in rats is very similar to that suffered by humans. The disease was provoked by means of injections of Zoledronate (ZOL; 35 mg/kg every 2 weeks), extraction of a maxillary molar, and insufficient vitamin D. The prevalence of osteonecrosis was: Vit D (--)/ZOL: 66.7%; Vit D (--): 0%; ZOL alone: 14.3%. Regarding PTH levels, when the sample was compared to a control group (Vit D (-), ZOL and combination of both) a statistically significant increase in PTH was observed in the Vit D (-), an increase in the ZOL alone group (although without statistical significance), and a significant increase in the combination of Vit D (-) and ZOL.

Further articles selected related osteonecrosis with osteomalacia. One made a case-control study using histomorphometric parameters by means of bone biopsies to analyze bone samples taken from patients medicated with bisphosphonates, 22 with osteonecrosis, and 21 without (20). In this study, vitamin D supplementation was an exclusion criterion. 77% of patients with osteonecrosis suffered osteomalacia compared with 5% of those who did not have osteonecrosis. Given that osteomalacia was found almost exclusively among patients with osteonecrosis, it can be said that osteomalacia represents a risk factor for osteonecrosis pathogenesis.

## Discussion

The bibliography reviewed contained very few studies that measured all four of the basic parameters this review set out to relate: PTH, Bisphosphonates, vitamin D, calcium and osteonecrosis of the jaw. Only one study (19) covered all four parameters, indicating a higher of incidence of osteonecrosis in rats. In all cases, vitamin D deficiency produced disorders in calcium and PTH levels.

Another study (20) that measured osteomalacia in patients treated with bisphosphonates, observed that 77% of patients with osteonecrosis suffered osteomalacia in comparison with 5% of control subjects without osteonecrosis.

An editorial (12) posed the question of whether the increase in PTH levels and hypocalcemia during bisphosphonate treatment in patients with metastatic breast cancer could be predisposing factors to ONJ.

In other articles reviewed, vitamin D and calcium supplementation was a basic inclusion criterion: - (17,18) - and also in the large randomized double-blind study performed using different antiosteoporotic drugs designed to test their anti-fracture efficacy (21-25).

A further three articles obtained contradictory results with regard to PTH as a predictive value of osteonecrosis but failed to contemplate vitamin D and calcium supplements in control cases (13-15).

In the five baseline studies carried out to test different antiosteoporotic drugs and their anti-fracture effects, calcium and vitamin D are administered to all participants. The antifracture efficacy of bisphosphonates could not be demonstrated in absence of adequate levels of vitamin D and calcium (21-25). Furthermore, the drug’s safety against hypocalcemia cannot be ensured unless there is an adequate supplementation as shown in the studies by (26-28) who described two cases of severe hypocalcemia in a hypothyroid patient and another with occult osteomalacia.

Generating further controversy (29), it was seen that a high percentage of the 38 osteoporotic, post-menopausal women treated with oral bisphosphonates, undergoing dental extractions, failed to meet the correct prescription criteria. It might even be said that diagnosis had been poor, given that only 6 subjects underwent adequate diagnostic tests.

According to the literature reviewed, (10) there is a generalized worldwide lack of vitamin D. Depending on the type of population studied and its cutoff point, the prevalence of the deficiency (< 20ng/ml) ranges between some 30% of young people and 80% of the aged in care, with 50-70% prevalence among the middle-aged (including adults and post-menopausal women) and elderly not in care.

It is evident that bone metabolism is directly affected by bisphosphonate administration. At the same time, this review observed a situation of great complexity regarding the balance of bone metabolism due to the high number of mediators involved. What is clear is the need to maintain optimal serum levels of the different mediators of bone metabolism, as much for the sake of the correct functioning of the bisphosphonates as the possible prevention of ONJ and other adverse effects.

The keeping of anamneses detailing daily vitamin D and calcium supplements in relation to bisphosphonate treatment could be of great importance in both, dental or medical practice, as this could help prevent ONJ. In cases where the patient fails to take these supplements and the clinician has to intervene, a prior appointment with the endocrinologist is advisable in order to establish the state of bone metabolism and so adjust vitamin D, calcium and PTH levels.

In any case, focusing on the potential risk of ONJ will permit the patient to make better use of bisphosphonate medication (30). The lack of unanimity as to the use of vitamin D and C supplements among different studies is one of the main constraints on this review’s findings, in addition to the low number of articles published to date. For these reasons, it is clear that further studies of these issues are called for within the different medical fields involved, given the multiple functions and the systemic implications that these four parameters can have. In this way, the relations between bisphosphonates and osteonecrosis of the jaw represents a complex field which could be investigated through a wide range of research approaches.
